# *Bartonella henselae* Infection: An Uncommon Mimicker of Autoimmune Disease

**DOI:** 10.1155/2013/726826

**Published:** 2013-01-17

**Authors:** Despoina N. Maritsi, Diagoras Zarganis, Zoi Metaxa, Georgia Papaioannou, George Vartzelis

**Affiliations:** ^1^Second Department of Academic Pediatrics, Athens Medical School, National and Kapodistrian University of Athens, 11527 Athens, Greece; ^2^Department of Paediatrics, MITERA Childrens' Hospital, 15237 Athens, Greece; ^3^Department of Pediatric Radiology, MITERA Childrens' Hospital, 15237 Athens, Greece

## Abstract

We present a case of a seven-year-old immunocompetent female patient who developed systemic symptoms mimicking an autoimmune rather than an infectious disease. The patient presented with rash, biquotidian fever, night sweats, and arthralgias. There was no antecedent history of cat contact. Investigations showed increased inflammatory markers, leukocytosis, thrombocytosis, hypercalcemia, and raised angiotensin-converting enzyme. Interferon-gamma releasing assay for tuberculosis infection was negative. Abdominal imaging demonstrated multifocal lesions of the liver and spleen (later proved to be granulomata), chest X-ray showed enlarged hilar lymph nodes, and ophthalmology review revealed uveitis. Clinical, laboratory, and imaging features pointed towards sarcoidosis. Subsequently, raised titers (IgM 1 : 32, IgG 1 : 256) against *Bartonella* confirmed the diagnosis of *B. henselae* infection. She was treated with gentamycin followed by ciprofloxacin; repeat investigations showed complete resolution of findings. The presence of hepatic and splenic lesions in children with bartonellosis is well documented. Our case, however, exhibited certain unusual findings such as the coexistence of acute ocular and systemic involvement in an immunocompetent host. Serological testing is an inexpensive and effective way to diagnose bartonellosis in immunocompetent patients; we suggest that bartonella serology is included in the baseline tests performed on children with prolonged fever even in the absence of contact with cats in countries where bartonellosis is prevalent.

## 1. Introduction

Cat scratch disease (CSD) is a common zoonosis in children caused by *Bartonella henselae* (*B. henselae*). Typical CSD, which occurs in 90% of cases, is characterized by low grade fever and subacute regional lymphadenitis following a recent history of cat contact. The disease has a self-limited course and usually resolves without the use of antibiotics. Apart from typical CSD, *B. henselae* has been recognized as a rare cause of fever of unknown origin (FUO) [1]. Nowadays, with the use of modern diagnostic tools the clinical spectrum of *B. henselae* disease has further expanded to include various additional clinical entities such as disseminated infection and septicemia, neurological, cardiovascular, ocular, and musculoskeletal manifestations [2]. We present a case of a seven-year-old immunocompetent female patient who developed ocular and systemic symptoms mimicking an inflammatory rather than an infectious disease.

## 2. Case Presentation

**Figure 1:**
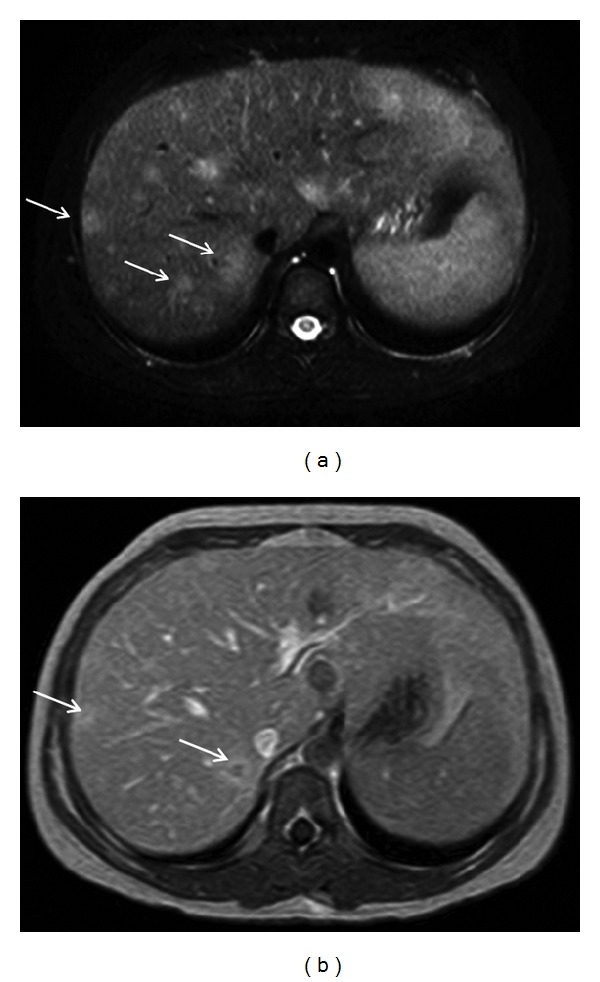
Abdominal MRI, axial images through the liver: (a) STIR sequence reveals multiple high intensity focal lesions (arrows) with indistinct borders due to perilesional edema in the liver; the lesions demonstrate peripheral enhancing rim in the T1W images after administration of IV contrast medium (arrows in (b)).

**Figure 2:**
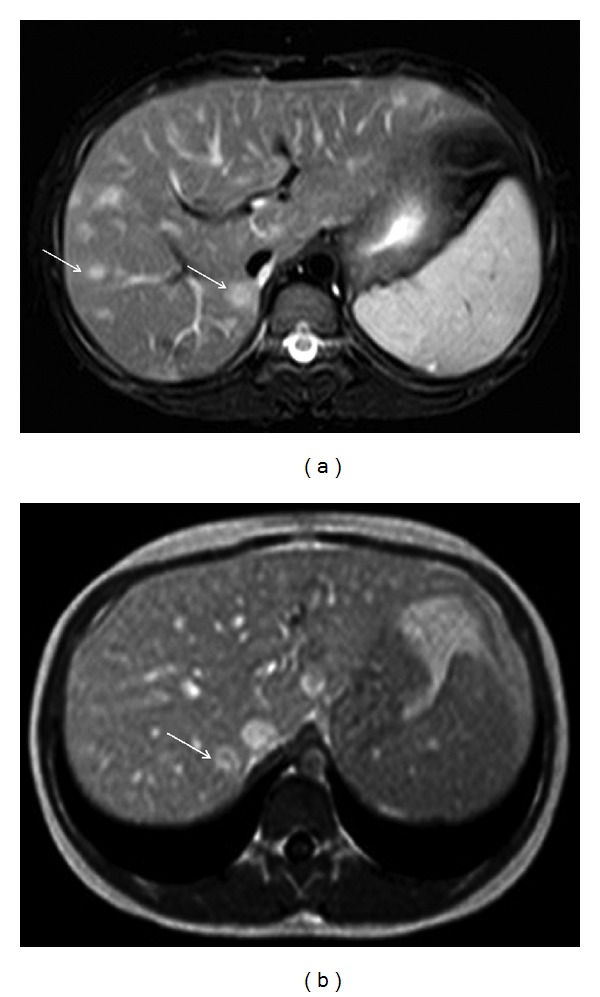
Follow-up abdominal MRI, axial images through the liver: the lesions (arrows) seen in Figure 1 present signs of maturity. Their margin is more distinct (STIR image in (a)), as edema has resolved, and the peripheral enhancing rim is better defined (T1W image after IV contrast administration in (b)).

The patient, offspring of an unrelated Caucasian couple with uneventful past medical history, presented with a three-week history of malaise, poor appetite, weight loss, biquotidian fever, night sweats, arthralgias, and an erythematous papular rash. Prior to this presentation parents reported an episode of unilateral conjunctivitis which lasted for three days, successfully treated with topical antibiotics. There was no antecedent history of cat contact; however the family lived in a rural area, and the child spent a lot of time playing outdoors. Investigations showed increased inflammatory markers ESR 86 mm/h (reference range (rr): 0–20 mm/h), CRP 8 mg/dL (rr: 0–0.5 mg/dL), leukocytosis (18000/mm^3^ (rr: 4000–12000/mm^3^)), thrombocytosis (650/mm^3^ (rr: 150–450/mm^3^)), mild hypercalcemia (11.8 mg/dL (rr: 8.5–10.5 mg/dL)), and raised angiotensin converting enzyme (ACE) (120 mg/dL (rr: 25–68 mg/dL)). Liver functions tests were moderately raised (AST: 98 IU/mL (rr: 25–46 IU/mL), ALT: 78 (rr: 15–35 IU/mL)). Interferon-gamma releasing assay test for tuberculosis (Quantiferon Gold in Tube test) was negative. Testing for ANA, anti-ds-DNA, ENA, anti-MPO, and anti-PR3 antibodies were negative. Abdominal imaging with ultrasound scan (USS) showed small hypoechoic lesions in the liver and spleen; subsequent magnetic resonance imaging (MRI) revealed multiple focal lesions in the aforementioned organs in keeping with granulomata (Figure 1), with associated lymphadenopathy. The chest roentogram showed mildly enlarged hilar lymph nodes; electrocardiogram and echocardiography were normal. Renal function tests as well as renal and urinary tract imaging did not show any abnormal findings. Ophthalmology examination showed unilateral grade 1 anterior nongranulomatous uveitis; the posterior segment was clear, intraocular pressure was normal, and fundoscopy was unremarkable. Musculoskeletal examination was normal. Clinical, laboratory, and imaging features strongly suggested sarcoidosis, and a liver biopsy was planned in order to confirm the diagnosis. However, raised titers (IgM 1 : 32, IgG 1 : 256 measured by indirect immunofluorescent assay (IFA)) against ex pointed towards the diagnosis of *B. henselae* infection. Polymerase chain reaction (PCR) performed on blood sample was negative for *B. henselae*. Blood cultures and serology for other causes of FUO (CMV, toxoplasmosis, *Toxocara*, and *Brucella*) were negative. Baseline screening for an underlying immunodeficiency including lymphocyte subpopulation analysis, IgG subclasses, and quantitative IgA, IgM, and IgE measurements was normal. She was treated with intravenous gentamycin for 7 days, followed by oral ciprofloxacin for 21 days. A combination of topical steroids and tobramycin for a total period of 6 weeks was used to control her uveitis. Fever and constitutional symptoms improved within 72 hours of commencing therapy; repeat clinical and serological investigations showed complete resolution of findings; IgM titers became negative after three months, while IgG titers peaked at three months (1 : 1024) and then gradually decreased over a period of six months. Repeat MRI showed calcification of the liver and splenic lesions calcified, but there was no central necrosis (Figure 2). Ophthalmology review held at monthly intervals for 12 months did not show any uveitis flares following termination of topical and systemic treatment.

## 3. Discussion

The commonest manifestation of *B. henselae* infection is cat scratch disease. Typical CSD is characterized by low grade fever and tender unilateral regional lymphadenopathy. In the majority of cases there is an antecedent history of direct cat contact. *B. henselae* has seasonal distribution from October to January. The disease course is often mild and self-limiting, rendering monitoring of the true incidence difficult to estimate. *B. henselae* is a small, aerobic, intracellular gram-negative pleoomorphic bacillus, microscopically identified by Warthin-Starry stain. The bacillus is very difficult to grow; hence culture is not routinely recommended. PCR in blood or tissue specimens is the most effective way to exclude bartonellosis; however this method lacks sensitivity (43–76%) [3]. On the other hand, serological testing is an inexpensive tool to diagnose *B. henselae* with 95% sensitivity and 98% specificity [4]. An IgM IFA titer of 1 : 16 or above is usually considered evidence for early infection. An IgG IFA titer of greater than 1 : 256 is considered evidence of current or past *B. henselae* infection. In the presence of a positive history of cat contact and relevant clinical findings, titers are reliable and sensitive leading to prompt diagnosis and initiation of appropriate treatment, thus avoiding unnecessary invasive investigations. Nevertheless, in cases where results are equivocal or in cases of immune deficient hosts, criteria have been proposed in order to reach diagnosis [5].

In few cases, however, symptoms and signs of the infection are constitutional and nonspecific. In a series of 146 children with PUO, *B. henselae* accounted for 5% of the events [1]. In the context of PUO, a variety of rare systemic manifestations have been described including endocarditis, osteomyelitis, and hepatosplenic, pulmonary, and central nervous system involvement [2], although infrequent eye manifestations include Parinauds oculoglandular syndrome, characterized by fever, unilateral conjunctivitis, and preauricular lymphadenopathy, chorioretinitis, retinal vasculitis, optic neuritis, panuveitis, and iridocyclitis [6]. In the majority of cases ocular bartonellosis follows a chronic course [7]; acute uveitis has also been reported [8] in the absence, however, of concomitant systemic inflammation. Other important infectious causes which could present with systemic and ocular involvement such as HSV_1_, leptospirosis, *Toxocara,* and tuberculosis, while histoplasmosis, toxoplasma, and CMV infections are not typically diagnosed in immunocompetent hosts. On the other hand, although rarely seen in children, the majority of small vessel granulomatous vasculitides could present acutely with systemic inflammation, ocular and organ involvement. Sarcoidosis is an idiopathic multisystemic inflammatory disease manifesting with noncaseating epithelioid cell granulomata in affected tissues. Classical sarcoidosis commonly presents with fever, malaise, lymphadenopathy, elevated inflammatory markers, and raised ACE. None of these findings, however, are specific for sarcoidosis, and tissue biopsy remains the gold standard for diagnosis. The mechanism by which ACE increases is the same either due to an infectious or an inflammatory cause, while the reason why this phenomenon occurs remains unknown. Epithelioid cells and macrophages in the granuloma produce ACE, but this finding is neither sensitive nor specific; nonetheless, it may assist in sarcoidosis disease monitoring.

The differential diagnosis of multiple hepatic and splenic focal lesions with features of peripheral enhancing rim and perilesional edema in children includes the wide spectrum of systemic, usually nonbacterial, infections, systemic autoimmune diseases and malignancies, predominantly of lymphatic origin. In this particular child, serial imaging confirmed the natural evolution of the lesions (from indistinct to better defined and eventually calcified on late imaging) which was unrelated to therapy and added additional value to the hypothesis that they represented granulomata.

The therapeutic approach for *B. henselae* infection is not standardized; it depends on disease severity and immune status. Typical CSD is a self-limited illness that resolves within 2 to 6 months and does not usually require treatment. Disease dissemination occurs in 14% of cases; therefore in patients with solid organ involvement and systemic inflammation 3-4 weeks of antibiotics are required [9]. Therapeutic agents which have shown satisfactory results include aminoglycosides, cefotaxime, and tetracyclines, while macrolides may be used in uncomplicated cases. Prevention is achieved by avoiding interactions with kittens, cats infested with fleas, and outdoor cats.

## 4. Conclusion

The presence of hepatosplenic lesions in children with bartonellosis is well documented. Acute uveitis caused by *B. henselae*, although exceedingly rare, has also been reported as an isolated finding; the case described is rare as it presents the co-existence of acute ocular and hepatosplenic involvement in an immunocompetent host in the absence of cat contact history. Serological testing is an inexpensive and effective way to diagnose bartonellosis in immunocompetent patients. We suggest that *B. henselae* serology is included in the baseline tests performed in children with FUO even in the absence of direct contact with cats. We also suggest an ophthalmology examination on children with constitutional symptoms attributed to *B. henselae*. Ocular involvement harbors severe consequences and requires prolonged use of antibiotics and close monitoring.
